# Conscientious Objection in Healthcare: The Requirement of Justification, The Moral Threshold, and Military Refusals

**DOI:** 10.1111/jore.12451

**Published:** 2024-03

**Authors:** Tomasz Żuradzki

**Affiliations:** Institute of Philosophy at Jagiellonian University in Kraków (Poland) and head of the Interdisciplinary Centre for Ethics at Jagiellonian University, where he leads the project BIOUNCERTAINTY funded by a European Research Council (ERC) Starting Grant

**Keywords:** conscience, conscientious objection, abortion, patient rights, healthcare law, conscientious objectors

## Abstract

A dogma accepted in many ethical, religious, and legal frameworks is that the reasons behind conscientious objection (CO) in healthcare cannot be evaluated or judged by any institution because conscience is individual and autonomous. This paper shows that this background view is mistaken: the requirement to reveal and explain the reasons for conscientious objection in healthcare is ethically justified and legally desirable. Referring to real healthcare cases and legal regulations, this paper argues that these reasons should be evaluated either ex ante or ex post and defends novel conceptual claims that have not been analyzed in the debates on CO. First, a moral threshold requirement: CO is only justified if the reasons behind a refusal are of a moral nature and meet a certain threshold of moral importance. Second, this paper considers the rarely discussed conceptual similarities between CO in healthcare and the legal regulations concerning military refusals that place the burden of proof on conscientious objectors. This paper concludes that conscientious objection in healthcare can be accommodated only in some cases of destroying or killing human organisms.

## 1 Introduction: The Requirement of Justification

In its application to the Polish Constitutional Tribunal (*Trybunał Konstytucyjny [TK]*) concerning the legislation on the conscience clause, the Supreme Medical Council (*Naczelna Izba Lekarska [NIL]*) wrote: “since it is commonly assumed that conscience is sovereign, then it is not known why a physician should justify his worldview in writing” (NIL 2014). The Tribunal dismissed this part of the complaint in its judgment of October 7, 2015. Even so, it agreed that “the purpose of keeping medical records is not to … record the philosophical and legal views of a physician in writing.” TK also maintained that the written justification for conscientious objection by a physician “should be of medical nature, and not serve to clarify the worldview of a physician or indicate a moral rule underlying his behavior.”^1^

Contrary to this judgment, this paper will show that the requirement to state reasons for conscientious objection in healthcare is ethically justified, legally desirable, and consistent with an important part of Christian moral tradition. It will challenge the dogma widely accepted beyond Polish law that, since conscience is “individual,” “autonomous,” and “sovereign,” the reasons behind a refusal to perform a given medical service cannot, by their very nature, be subject to any evaluation or regulation (Wicclair 2011). This dogma is grounded in the assumption that there is something like individual moral integrity that grounds a pre-institutional and indefeasible right to conscientious refusal. This argument assumes that the public expression of a worldview is a constitutive practice for any judgment of conscience. It also shows that the lack of a requirement for a justification exposes patients to the risk of bearing additional costs, even in situations where there is no need to protect the integrity of a physician’s conscience.

Building an argument mostly on real-life cases from Poland, where the legal right to conscientious objection has lexical priority^2^ over any other right (as stated in TK 2015) and where the CO clauses have been significantly overused in practice (see examples below), this paper will defend an interpretation of the justification requirement, according to which invoking the conscience clause in healthcare is ethically permissible only when all of the following conditions are met: (1) authenticity: there is no reasonably plausible evidence that the judgments are not genuinely adopted by a physician; (2) relevance: the judgments of conscience are not based on false beliefs about current scientific knowledge or false beliefs about the circumstances of the case or false beliefs about the content of legal, ethical, or religious norms; (3) moral threshold: the normative judgment forming the basis for invoking the conscience clause is of a moral nature and exceeds some threshold of moral significance or importance. This last condition has not been analyzed in the literature on CO. The position presented here, on the one hand, significantly extends the so-called reasonability view (Card 2007, 2011, 2014; LaFollette and LaFollette 2007; Kantymir and McLeod 2014; Marsh 2014), but on the other hand, it differs from these views (Savulescu and Schuklenk 2017; Schuklenk and Smalling 2017), which see conscientious objection in healthcare as never justifiable. This paper starts with some brief introductory remarks about the theological sources of valuing individual judgments of conscience before introducing the requirement of a moral threshold. Finally, it develops the similarities between the problem of the medical conscience clause and conscientious objection by conscripts and professional soldiers in military contexts.

## 2 Overvaluing Conscience and the Limits of Legal Protection

The purpose of introducing a legal form of conscience clause was to protect some people against performing acts that contradict their judgments of conscience or—as often specified in literature—violate their moral integrity. Such integrity can be understood as the relation between accepted principles and performed or expected acts (Childress 1979; McFall 1987). Many assume that it is wrong to act deliberately against one’s moral beliefs, and it would also be wrong, at least prima facie, to make someone else act against her moral beliefs, no matter the content of her moral beliefs (Brock 2008; Wicclair 2011).

The recent judgment of the Polish Constitutional Tribunal that holds that “the right to conscientious objection should be considered a primary right in relation to its limitations” (TK 2015) is an excellent example of the overvaluation of conscience in legal documents. This somewhat unclear fragment is probably supposed to mean that any other value must never limit the freedom of conscience (as a constitutional value) and that it is not even possible to compare or weigh any other values competitive with freedom of conscience. In other words, the value of the freedom of conscience has lexical priority over any other value. In one of the most surprising fragments of the judgment, the Tribunal states that “restriction of the freedom of conscience cannot be subjected to the proportionality test, due to the inability to determine which constitutional values the legislature intends to protect at the expense of physicians’ conscience” (TK 2015). Therefore, the Tribunal assumes that the right of a patient to obtain legal and guaranteed medical services, for example to abortion, does not constitute such a value (abortion, even in Poland, in the extremally rare cases when it is legal, is treated by law as a guaranteed medical service).^3^ What is more, the introduction by the Tribunal de facto of an “on-demand conscience clause” for physicians, without any external control, leads to the necessity to respect such statements even if there would be no breach of the moral integrity of a physician because the judgment of conscience would turn out to be, for example, inauthentic, irrelevant, or would not be of a moral nature at all. In such a case, we would not protect any values, and the patients would bear losses arbitrarily.

At first glance, it may seem that the Tribunal’s view on conscience (in its legal sense) stems from a theological understanding, for example that conscience is hidden, private, and inaccessible to outside engagement. Some documents of the Catholic Church may suggest such an interpretation, for example the fragment of the pastoral constitution *Gaudium et Spes* describing conscience as “the most secret core and sanctuary” of a person (Paul VI 1965). However, this understanding contradicts other interpretations of Catholic teaching in which judgments of conscience are understood as responses to objective and publicly available reasons. One common interpretation of this view that frequently appears in a wide range of contemporary debates about CO refers explicitly or implicitly to Thomistic thought. It assumes that believing that what one is doing is morally good and acting in accordance with one’s conscience is a necessary but not sufficient condition for morally good actions because, under this interpretation, a judgment of conscience may be false in two ways: “first, when a particular action or kind of action is judged incorrectly to violate or follow from some universal moral principle; second, when one’s syllogism appeals to the wrong universal moral principle by falsely identifying the most morally salient aspect of the action” (Chanderbhan 2016, 3).^4^ Obviously, we have an obligation to follow correct judgments of conscience. But what of erroneous ones? According to Aquinas, conscience always binds, regardless of whether someone’s action is objectively good, evil, or indifferent (Hoffmann 2012). The justification for this view is that by choosing the opposite of what someone’s conscience dictated, one would choose to do something one believes to be evil. This controversial view leads to the approach defended by some commentators that the right to CO should not be limited in any way: “the medical professional must demand that his or her serious judgments about what is (or is not) good for the patient be respected, just because acting in good faith is a necessary (although not a sufficient) condition for any good medical conduct and any real care about patients” (Głowala 2016, 26).

However, Aquinas distinguishes between vincible and invincible ignorance, and only this second type, which concerns ignorance about the facts of the situation, may excuse someone-and only if no negligence is involved. In contrast, if a judgment of conscience results from vincible ignorance (which is sinful itself), that is, negligence or ignorance of the relevant moral principles, one is culpable for following one’s conscience, although it is still binding. Moreover, Aquinas assumes that it is everyone’s obligation to know the moral law, so this type of ignorance is always vincible.

This paper defends the view that not every judgment of conscience to which someone is committed deserves protection. It assumes that although this conception should primarily fit modern secular institutions, it can also be accepted by those who refer to the Catholic interpretation of conscience understood as the ability to recognize objective norms of morality that may be publicly expressed and defended. The view defended in this paper may help to solve the most problematic aspect of the Thomistic approach, that is, that one may be culpable for following one’s judgment of consciousness stemming from vincible ignorance. The next section discusses some real and hypothetical examples that may help to develop the limits that legal protection of properly understood moral integrity should have.^5^

## 3 Criteria for Conscientious Objection in Healthcare

### 3.1 Authenticity

The first criterion concerns the requirement of authenticity. Let us imagine that a physician refuses to perform certain medical procedures in a public health care institution, based on the conscience clause, but performs them in other institutions or at his private practice or that a conscript refuses military service while being a citizen of a country in which it is mandatory, but works in a private military company on his own (for example, Academi, the former Blackwater) and performs duties typical for soldiers. These instances would be sufficient evidence of the inauthenticity of judgments of conscience. In these cases, the issue is clear: we are not dealing with the integrity of conscience at all—no matter whether one intentionally lies about his judgment of conscience or is simply confused about his own judgments. Some authors appeal to a similar standard, arguing that if someone’s judgments are incoherent (for example, “X is permissible” and “X is not permissible”), at least one of them must not only be irrational, but it also cannot warrant accommodation (Meyers and Woods 1996).^6^ By recognizing this as a type of conscientious objection, we would protect only some nonmoral interests, including financial interests, but not the integrity of their conscience.

It is worth noting that the above example of a physician is not only one of many merely hypothetical examples discussed in the bioethics literature on CO, but it strictly resembles some actual cases, in particular, concerning abortion. For example, it seems that in Poland, the requirement of authenticity may be massively violated because of the structure of legal regulations and nonlegal incentives. This claim cannot be tested “empirically,” but below, comparative and historical analyses of law, together with some supplementary anecdotal evidence, validate this hypothesis.

Abortion in Poland is legal only if at least one of the following conditions is fulfilled (Sejm 1993): (i) there is a justified suspicion that the pregnancy is the result of a crime; (ii) the pregnancy endangers the woman’s life or health; (however, in this second case, physicians are not allowed to mobilize the conscience clause (Sejm 1996); The third condition, (iii) that prenatal tests or other medical evidence shows that there is a high probability of a severe and irreversible fetal impairment or incurable illness endangering its life, was judged unconstitutional by the Polish Constitutional Tribunal on October 22, 2020 (TK 2020; the legal status of this institution has been contested since 2015; see Sadurski 2019).

The official abortion rate, even before October 2020, was extremely low: in 2019, there were 1110 legal abortions (0.13 per 1000 women aged 15–44), out of which 1076 abortions were performed because of the third condition, which has since then been declared unconstitutional. Since in previous years, 95%–98% of all abortions were performed because of the third condition, it may be expected that in Poland, in the coming years, there will be between 25 and 55 legal abortions annually. Moreover, the official number of legal abortion procedures performed in public hospitals in Poland fell by 99% during the early 90s: from 160,000 cases in the late 1980s to about 160 yearly in the late 90s. The percentage of abortions per 1000 women is much lower than in any other European country, with the exception of Malta. For example, in Italy in 2010, the same rate was 10.0, in the US in 2011—16.9, and in England and Wales in 2015—16.0. Thus, it is easy to suspect, as many Polish NGOs do, that abortion rates in Poland are much closer to the Italian rates, but most procedures are performed in the gray area (Chełstowska 2011). Moreover, in Poland, according to official ministry reports, some parts of the country had no official abortion providers before October 2020 because of the scale of CO refusals.^7^

Obviously, one could wonder in these circumstances whether the reasons of all physicians in these regions for CO (before October 2020) were authentic, that is, whether all physicians in this region believed in the full moral status of the fetus or rather only use the CO clause for their own professional convenience and the fulfillment of the expectations of their supervisors. This kind of top-down pressure is openly and frequently expressed by state officials. For example, Konstanty Radziwiłł, an influential physician, the previous president of the Polish Supreme Medical Council between 2001 and 2010, the Ministry of Health between 2015 and 2018, and now the voivode of Masovian Voivodeship, explicitly claims that he would refer to CO, not only in the case of abortion, but also in the case of prescribing a morning-after pill to a rape victim (RadioZET. pl 2017).

### 3.2 Relevance

The second example concerns the requirement of relevance: let us assume that a physician mistakenly believes, despite readily available and reliable information, that a given drug or procedure causes the death of an early embryo when it only prevents fertilization (Card 2007). She refuses to prescribe the drug, claiming that her conscience does not allow her to participate in the destruction of human embryos. In this case, a physician is mistaken about empirical facts.

It is worth noting that it is also possible to have false beliefs about the content of legal or ethical norms (including the professional codes), which is more problematic. As an example, let us assume that a Catholic physician falsely believes that an indirect abortion (that is, treating a life-threatening pathology in such a way that the treatment inadvertently leads to the death of the fetus) may never be permissible within Catholic doctrine. This type of abortion is commonly treated as permissible even within Catholicism since it may be justified by the principle of double effect (PDE), which assumes that the intended aim is to remove not the fetus but some biological material (for example, a cancerous but gravid uterus or a placenta) that is treated as a real threat to the pregnant woman’s life and also the cause of the fatal threat to the fetus.

A similar case of false beliefs about the content of legal or ethical norms may concern other spheres of life, particularly the military. Let us assume that a conscript refuses military service by claiming that soldiers are required to direct military operations against non-combatant targets, which—as he claims—lies in contradiction to his conscience. In reality, he is wrong: all rules of engagement accepted by Western countries explicitly require soldiers to follow international humanitarian law in combat situations and forbid such targeting, although they allow the destruction of such targets, but only unintentionally, and only when civilian or non-combatant losses are not excessive in relation to the “concrete and direct military advantage anticipated” (Protocol I 1977). This case will be discussed further in section four.

However, it is sometimes more difficult to establish the content of norms, particularly if some norms are contested even within a religious or moral doctrine. For example, the Catholic tradition has surprisingly not reached a satisfactory consensus on the permissibility of performing an abortion in life-threatening emergency circumstances when the pregnancy itself endangers a woman’s life and before the fetus is viable (for example, cases of pregnancy with pulmonary hypertension).^8^ The controversies concern both the very permissibility and possible justifications for this practice. Some authors suggest, contrary to the mainstream teaching, that direct abortion should be permissible by Catholic moral teaching because of the principle of choosing the lesser evil, which states that in a situation of choice between two or more evils (in this case, the death of one person or the death of two), one is obliged to perform the least wrong action (Prusak 2011; Sulmasy 2007). I have argued elsewhere that claims for conscientious exemption might be justified only by judgments that the representatives of a relevant tradition commonly recognize (Żuradzki 2016 see also Ciszewski 2021).

In the case of an inauthentic claim of conscience, there is actually no clash between judgments of conscience and the provision of a given medical or social service. In the case of irrelevant judgments, there would be no collision if the person concerned acquired a correct judgment or got rid of the false one. In both cases, the aim of the requirement to reveal and explain reasons for CO (for example, in the form of a written justification presented to a special commission) would detect only whether or not there is any collision between a medical service and a judgment of consciousness. In this sense, revealing the reasons for CO serves to check the authenticity and relevancy of the judgments of conscience (Meyers and Woods 1996, 2007; Weinstock 2014). Robert F. Card, who defends similar requirements in the case of the medical conscience clause, defines them cumulatively as the reasonability view (Card 2014). In his understanding, this view includes, among other things, what has been described here as the authenticity requirement and, partly, the relevancy requirement (intrinsic factors), as well as several additional (extrinsic) conditions, which are not discussed in this text as they are non-controversial and enshrined in either law and/or the code of medical ethics: the requirement to avoid unnecessary harm to patients, the requirement to avoid being guided by self-interest, the requirement to avoid discrimination, *etc*.

The view defended in this paper expands Card’s reasonability view in two aspects. First, this paper assumes that the requirement of relevancy concerns not only the empirical data and circumstances of the case but also the content of legal or ethical norms. Second, and more importantly, these two requirements, authenticity and relevance, are not sufficient in this interpretation. We need also the moral threshold requirement: that a judgment of conscience, on which basis someone refuses to perform a medical service, is of a moral nature and exceeds some threshold of significance or importance.

It is important to not mix together two distinct issues in this context: (1) whether the basis of the person’s objection is morally plausible or it derives, instead, from a judgment that is morally perverse or pernicious (and in this second case, CO would be rejected in the light of Card’s extrinsic factors or my first two requirements together with the uncontroversial regulations of the code of medical ethics); and (2) whether the ground of the person’s objection is or is not morally important enough (CO would not be rejected on the reasonability view or my two first requirements, but on my moral threshold requirement).

Here is a real example that depicts the first case (Siedlecka 2016). A physician from northern Poland refused to hand over information about health conditions to a homosexual woman (legally single, since homosexual marriages are not allowed in Poland) after recognizing that the information would be used in an adoption process. The physician grounded her initial refusal only on the statement that the adoption by a homosexual person “would be wrong for a child,” although finally, the physician got a rebuke from her supervisor and the patient ombudsperson. This example resembles fictional cases described in the bioethics literature about physicians who refuse to perform a given service in relation to persons of a particular gender, religion, or race (Wicclair 2011). Although in cases like these, one does not have to doubt the authenticity of the beliefs of the physician, the judgment of conscience violates the relevance requirement (or Card’s extrinsic conditions) because it goes against the standard codes of medical ethics that explicitly prohibit discrimination based on “race, religion, nationality, political views, financial status and other” (*Naczelna Izba Lekarska [NIL]* 2003).^9^ It is commonly accepted in such codes that even if a physician may refuse to provide *some* treatments referring to CO, she may not apply CO to refuse to treat *some* patients. This approach may also exclude the possibility of refusing certain medical services due to a critical evaluation of the goals or choices of the patient (for example, some bioethicists discuss a hypothetical example of a physician refusing the further treatment of a patient who rejects the next chemo treatment series, see Wicclair 2011).

### 3.3 Threshold

Therefore, the question is how to formulate the moral threshold requirement and how to distinguish the moral grounds for conscientious objection from grounds that do not have a moral nature and/or are not sufficiently morally important. Some subjectivist accounts suggest that a valid judgment of conscience could depend merely on evaluating the beliefs and values of the individual claimant (Billingham 2017). According to these accounts, the importance is determined by the level of obligatoriness (for example, a strictly obligatory avoidance of abortion *vs*. a non-obligatory cross necklace in Catholicism) and centrality for someone’s moral integrity (a cross necklace in Catholicism may still be central for someone’s faith, even if not obligatory, see ECHR 2013; Maher 2014). The approach defended in this paper is different because it argues that an individual’s evaluation of the importance of a judgment of conscience may be examined only within the canon of a relevant, comprehensive doctrine, which is, by its nature, a social concept.

My approach relies on a commonly accepted distinction between moral and nonmoral reasons^10^ (although some authors believe that the distinctiveness of morality cannot be established prior to substantive inquiry into the content of moral reasons, see Dorsey 2016, and some others criticize reasons pluralism, which is a view that some reasons are distinctively moral while others are not, see Forcehimes and Semrau 2018). In particular, recent literature in moral psychology and metaethics commonly distinguishes moral from nonmoral (for example, conventional) norms and reasons (for an overview see O’Neill 2017). This first sphere is often treated as independent because moral norms are “not derivative of the verdicts of any other domain” (Dorsey 2013, 132). For example, Victor Kumar, who argues that moral judgments are a natural psychological kind that plays an essential explanatory role in psychological generalizations, summarizes psychological research on this topic: “the human cognitive system is organized in such a way that the four features have a nomological tendency to cluster together” (Kumar 2015, 2896). These four features are the following: seriousness (moral violations are treated as more serious), generality (moral judgments are to apply to any agent, also in other places and times), authority-independence, and objectivity (that is, moral judgments are conceptualized by people as objective, although this does not imply that there are objective moral properties). Some normative judgments do not share all four features, but they are atypical moral judgments. According to this approach, morality is treated as a social domain—it concerns only how to get along with others and not how to live one’s life or what moral duties one has with regard to oneself (so it is analogical to a classic distinction between morality and ethics, see Williams 1985). It is a reason why, when asked about the justification of some moral judgments, the participants of psychological research usually refer to the harm that moral violations may cause to others, to violations of others’ rights, or to injustice (Kumar 2015).

In the case of the threshold requirement, generality and objectivity seem particularly important. This understanding of moral judgments stipulates that a particular judgment serving as the basis for the CO exemption: (1) involves reasonable hope that it will be adhered to by other persons who find themselves in relevantly similar circumstances, regardless of their religious affiliation or moral views (this is not to say that one must seek to change others’ views or the law: we are constantly accepting that others may be morally mistaken, and many commonly entrenched moral judgments have no corresponding legal sanctions); (2) is formulated in terms of objective reasons about what ought or ought not to be done in a given situation that is intelligible to, even if not shared or endorsed by, all stakeholders.

Thus, at least in my understanding, the moral threshold requirement contradicts views that allow for CO on the basis of merely “irreducibly religious” grounds, namely, normative judgments that are rooted not in moral judgments of what is right and wrong but rather in the “requirements through which the believer evinces her *identification* with a historically extended community of believers” (Weinstock 2014, 14). It has been argued that accommodating “irreducibly religious” reasons that cannot be translated into moral vocabulary may have beneficial pragmatic effects on healthcare institutions. However, my examples show that grounding a judgment of conscience solely in the sense of identification with some community is not sufficient to be the basis for the CO exemption. For example, for some people, female genital cutting may represent a profound sense of identification with some communities, but it is not a reason for others to respect this judgment and the practice based on it. The same applies to physicians: judgments based solely on a sense of identification (for example, with religious groups) do not deserve special protection in healthcare.

Let us see the threshold requirement applied to examples: if someone actually recognizes the personal status of a human embryo or fetus (no matter if a religious or secular morality inspires her views), she will claim that other normative views—which do not assume such a status—are somehow wrong or mistaken and will hope that everyone should treat embryos or fetuses as if they had full moral status (even if she is not a pro-life activist). It is not because she wants to tell other women how to live *their* lives but because she believes that abortions kill *other* human beings. What makes these judgments genuinely moral is their intelligibility to others and reasonable expectations that they apply to everyone, no matter if they share a sense of identification with some community, for example, prolife activists or Catholics. This sense of identification may psychologically enforce these judgments but, in my interpretation, may not be the sole ground for holding them. Genuine moral judgments do not depend on a particular practice in a society: the fact that a physician lives in (and identifies with) a society where abortion is almost illegal or is officially treated by some authorities (politicians, clergymen) as highly immoral, does not constitute, as such, sufficient ground for holding a judgment, because genuine moral judgments are, as one scholar summarizes, “essentially practice-independent” (Southwood 2011). Interestingly, it would be much harder under this interpretation to justify a conscience judgment that it is impermissible for a healthcare practitioner to aid a person in dying. In opposition to the abortion case, it seems that in the case of medical aid in dying, one indeed tries to enforce one’s views on how to live one’s life or what moral duties one has with regard to oneself.

In contrast, a follower of Judaism who circumcises newborn boys cannot reasonably expect this from other people who do not follow his religion (that is, cannot expect that they do it because of religious reasons). The same concerns a follower of Islam who considers particular outfits or headgear to be proper or a Catholic who has specific beliefs about the appropriate goals of sexual intercourse which must always be open to the transmission of life and unitive (and that these two features are inseparable).^11^ This last example may be particularly important since many physicians in Catholic countries invoke CO and refuse to prescribe birth control that only prevents fertilization (but does not prevent the implantation of a fertilized egg). In such cases, they cannot claim that prescribing birth control is equivalent to killing (or even letting die) but only that they consider contraception itself to be immoral (the next subsection discusses a more complicated situation of uncertainty about whether a birth control pill may also prevent implantation). And this must be so because of their views on morally proper goals of sexual intercourse.

At first glance, it may seem that contrary to the above claim one can argue that this is an eminently moral issue for those Catholics opposed to contraception and that this stance has all the universality and objectivity ascribed to what is distinctly moral. However, there are serious reasons not to accommodate CO refusal on such justification. In contrast with abortion, where a pro-life physician—if forced to do an abortion herself—would indeed do something morally wrong in her own eyes (after all, she would intentionally kill a fetus), it is not the same in the case of merely “forcing” someone to prescribe contraceptives. In this case, a physician does not do anything morally wrong himself (in this sense that no one is, for example, harmed or wronged by his act), one only makes it easier for a woman (or a couple) to act in a way that is morally wrong in the physician’s eyes, and his action is neither necessary nor sufficient for what he thinks is a morally wrong event. After all, the couple may have “sinful” sex (that is, not “open to the transmission of life”) even without his prescription. In such a case, his act does not belong to the realm of morality (as defined above) but rather to ethics; that is, it violates the physician’s view about someone’s proper life conduct. In this respect, the situation of CO in the case of contraception is crucially different than CO in the case of abortion.^12^

Practically, it means that conscientious objection can only be met in some cases of abortion.^13^ Referring to the conscience clause in cases of prescribing contraceptives, including “morning after” pills that prevent fertilization, would not be acceptable because it is a sectarian view on the proper goal of sexual relations that mainly aims at identifying a believer with a community of co-believers.

Finally, it is worth distinguishing my views from two other approaches: public reason and that of the impartial observer. First, the Rawlsian “public reason” justification (that is, rules that all reasonable citizens would endorse) is an intersubjective and idealized practice of argumentation used where some person or group exercises coercive power over another person or group: “public justification is not simply valid reasoning, but argument addressed to others: it proceeds correctly from premises we accept and think others could reasonably accept to conclusions we think they could also reasonably accept” (Rawls 1993, 465). Some authors have defended this approach in the case of CO and require an objector to not only cast their objection in terms of public reason but also to show how those public reasons meaningfully connect with their sincerely held comprehensive conceptions (McConnell and Card 2019). In contrast with this interpretation, my approach does not require all reasonable citizens to *endorse* all justified CO claims. It is enough that the CO claims are broad enough to be generalizable and are formulated in terms of objective reasons.

Second, the view defended in this paper should also be distinguished from the conception of an impartial observer initially proposed by Adam Smith. In the case of CO in healthcare, the view assumes that claims of conscience are reasonable— and deserve being respected—”insofar as they approximate moral truth as determined from the standpoint of an impartial spectator” (Ben-Moshe 2019, 408). Thus, medical practitioners must provide the reasons for their conscientious objections that would be endorsed by an impartial spectator. This requirement should guarantee that their “claims of conscience are true, or at least approximate moral truth to the greatest degree possible for creatures like us” (Ben-Moshe 2019, 404). In contrast with this interpretation, my approach does not assume any metaethical view about the truth value of normative statements. Moreover, in some crucial cases, this approach resigns from its high aspirations and assumes that for example CO in the case of abortion should be respected not because it is true or at least represents approximate moral truth but because an impartial spectator would invoke epistemic humility about the moral permissibility of abortion (Ben-Moshe 2019, 405).

## 4 Conscientious Objection in the Case of Conscripts and Professional Soldiers

This section highlights the similarities between CO in healthcare and the regulations concerning military refusals, including an emerging practice of granting the right to selective CO status to professional soldiers that places the burden of proof on a petitioner for CO status. The main similarity between conscripts and physicians seems to be as follows: some of the conscripts refuse to participate in institutions that use violence and approve of the possibility of killing other people. They do not agree that reasons related to safety (self-defense, defending other people, protection of territory, etc.) are sufficient to justify the acceptability of killing or using violence, even when they are perfectly legal. Because the most popular case of applying the conscience clause by physicians is abortion, a simple analogy would state that some physicians consider fetuses to be entities with full moral status, and various ethical or legal considerations that allow abortion do not constitute, in their opinion, a sufficient reason to justify the abortion morally. So, the conscience clause in healthcare has its legal equivalent in the provisions concerning alternative military service for conscripts (these laws are still in force in many countries, but since the abolition of compulsory military service, they have been virtually defunct). In Poland, for example, the law exempted persons whose “religious beliefs or moral principles do not allow them to perform this service” from the obligation to perform military service. However, a person wanting to receive an exemption had to provide:

(1) a statement of their religious beliefs; (2) an indication of a basis found in the religious doctrine, which excludes the permissibility of performing military service, as well as a demonstration of a genuine link to the professed religious doctrine or an indication of one’s moral principles, which contradict the obligations of a soldier performing military service. (Sejm 2003).

Polish courts that rule on the basis of this provision stressed that the need to reveal one’s beliefs is not at odds with the constitutional right to privacy (understood as the right not to be forced to reveal your worldview), because it is the conscript who voluntarily undertakes not to perform a universal obligation, and without revealing those beliefs, it could not be determined whether they actually prohibit him from performing military duty (WSA 2008; ECHR 2011).

What is more, in recent years, there have been interesting academic discussions on the acceptability and understanding of selective conscientious objection in the case of professional soldiers. Some countries, in practice, have also begun to allow for conscientious objection in this case (Minear 2014). The United States is a particularly interesting case: in the years 2002–2006, as many as 425 persons applied for objector status, and in 224 cases, the matter was decided positively—despite the fact that the US military was completely professional and signing up for the military involved explicitly acknowledging that one was not a conscientious objector defined as: “a firm, fixed, and sincere objection to participation in war in any form or the bearing of arms, by reason of religious training and/or belief” (US Government Accountability Office 2007).

Two features of the law of military conscientious objection should be highlighted here: the requirement of generality and the requirement of anteriority. The first matter is that exemption from military service is possible only in cases where religion or the advocated principles “contradict the obligations of a soldier performing military service.” This means that the basis for exemption must lie in the objection to the performance of military service itself rather than participating in a particular armed conflict (US Supreme Court 1971).

There are pragmatic reasons for this view: it is easier for commissions to check the membership of a particular religious group than to assess the merits of arguments against a given military conflict. However, it does lead to quite a paradoxical result: pacifist positions in a strict sense, that is, ones that teach that no one should ever use force, even in self-defense or to prevent the use of force, are not philosophically credible (McMahan 2010). Therefore, some scholars have challenged the legitimacy of the selective exclusion of conscientious objection in the case of conscripts in recent years and have even called for the possibility of invoking conscientious objection in the case of professional soldiers who consider a given war unjust (Ellner, Robinson, and Whetham 2014; May 2012).

In the case of a medical conscience clause, there are also discrepancies concerning whether a physician should only have the ability to invoke a general or also selective conscientious objection. The recent judgment of the Polish Tribunal made these provisions similar to the regulations concerning conscripts by explicitly excluding the possibility of selective conscientious objection in the case of physicians. Such an interpretation—although it makes managing health care facilities easier—is problematic from a philosophical point of view, in the same way that it is problematic in the case of conscripts, because unconditional opposition to performing, for example abortions, is not an obviously defensible view, since one may believe that some abortions are permissible while others are not. It is easy to imagine a physician who recognizes that fetuses have full moral status and thus believes abortion to be generally wrong but who accepts that it might not only be permissible when the continuation of pregnancy seriously threatens the life or health of the pregnant woman but also when a pregnancy is the result of a rape, that is, when a woman has not done anything to lose her right to bodily autonomy.^14^ Nevertheless, she refuses to perform it in the case of genetic defects of the fetus, for example.

The second issue is the requirement of anteriority, which in law applies to both conscripts and physicians. Trybunał Konstytucyjny (TK) clarified the anteriority requirement in the case of physicians, stating that the notification of invoking the conscience clause should be “a priori, addressed to one’s superior, in principle, at the time of establishing the employment or service relationships, or possibly—during its course—when, as a result of a change of beliefs, a physician wants to refrain from performing services that they could perform earlier in line with their own conscience” (TK 2015). This clarification has very interesting features. First, it allows a physician to change their views at any time and disengage themselves from performing certain services. Second, a one-time declaration prevents a physician (or at least prevented her before October 2020) from being confronted with the need to refuse to perform certain services for specific patients, as they are simply not directed to that physician. According to the TK, this is one of the reasons why an obligation to provide a referral to an alternative physician or clinic cannot be prescribed to a physician who objects to a procedure on conscientious grounds (this judgment results in a situation where no person or institution in Poland has a duty to inform a patient where she can perform some procedures that are legal and covered by the state insurance). Third, prior notification of the employer on the decision to not perform certain services does not require any justification on the part of the physician and cannot be challenged by anyone.

As can be seen, the differences between the current legal regulations on conscientious objection in the case of physicians and conscripts are significant. On the example of the relevant Polish legal regulations, unlike conscripts, a physician: (1) never has to disclose their religious or moral views that form the basis of conscientious objection; (2) never has to explain why their worldview would prevent them from performing a given service; (3) nobody can verify or control their prior statement on the refusal to perform medical services; (4) may submit a refusal to perform a given type of services at any time.^15^

In the case of conscripts, it was assumed that the burden rests with them to demonstrate authenticity or relevance before a specially appointed commission. Is the situation of physicians and conscripts or soldiers conceptually similar enough to make the medical conscience clause similar to the military one? Yes, and it seems there are no good reasons for it to be different in the case of physicians and for their statements to be evaluated ex ante by such a commission or ex post by a court. Some argue that—at least in the case of certain countries (for example, the United Kingdom)—calling physicians who invoke CO before a special commission would not be a good idea because “the number of false positives would be very small, and not enough to justify the cost of running the tribunals” (Cowley 2016, 70). However, it seems that this is an issue that depends on the social context, and in other countries, like Poland, this type of argument is not sound.

One of the arguments against equating the situation of conscripts and physicians is that refusal in the case of conscripts affects a general obligation that is similar, for example, to tax obligation. Hence, one might argue that the reasons behind refusal should be more carefully examined in the case of conscripts than in the case of physicians who refuse to perform specific services over which they have a monopoly (for arguments against CO referring to public cartels, see Cholbi 2018). This argument, however, is double-edged: it could be argued that it is physicians who are obliged to provide more precise justification behind the refusal because they have undertaken their profession voluntarily and chose a specialization with full knowledge of what type of services they will have to perform (or at least the argument defended in this paper would concern these procedures that were legal when they entered the profession). In contrast with conscripts, no one forced them to choose their profession, nor are there any obstacles to resigning or changing their specialization at any given moment. Moreover, they have also embraced role-based duties, including providing legally sanctioned treatments unless there are important moral considerations in conflict, and they receive rich goods from society (salary, status, state-funded education, *etc*.). It does not matter (at least in the European context) whether a physician is a public or private employee since this paper concentrates on procedures funded by the public provider (no matter if in the public hospital or through a private contractor) and does not consider CO in the case of private, for-profit corporations which offer on-demand health services (West-Oram and Buyx 2016). In contrast, a vast majority of conscripts did not choose military service voluntarily, nor can they quit being soldiers or refuse service at a time of their choosing. So the status of CO in healthcare is perhaps not as similar to the case of conscripts but much closer to the case of professional soldiers who have voluntarily entered their profession, and at some point, changed their minds about the permissibility of their actions. If one agrees that the reasons behind such a refusal should be more carefully examined in the case of professional soldiers than conscripts, a fortiori one should also accept the strict examination of physicians’ reasons for CO status.

Moreover, one could argue against comparing the situation of conscripts or soldiers and physicians by considering that the scope of duties transferred to others is smaller in the case of physicians than in military personnel. Except for some cases of conscription during times of war, the situation is the opposite, and the consequences of not enlisting are felt less directly by the military or government. When physicians who have a monopoly on medical services refuse to perform a procedure, it makes patients’ access to guaranteed medical services difficult or sometimes even impossible and causes other physicians to have more work (I understand “guaranteed medical services” as services that are free of charge for all patients with standard public insurance coverage, so in Poland, it covers abortions in these rare situations when legal).

Such a transfer of costs onto others was particularly visible in the case of abortion in Poland (before October 2020). It was also noticed in some judgments of the European Court of Human Rights (ECHR), which ruled that the Polish government does not provide effective mechanisms for obtaining an abortion, even in these very rare cases when it is legal, for example in the famous case of a 14-year old girl from Lublin, whose pregnancy was the consequence of a criminal act (ECHR 2007).

In contrast, refusing military service at a time of peace in a country where conscription is universal (for example, today’s Israel) does not significantly increase the responsibilities of the remaining conscripts (because everyone is conscripted anyway) and does not diminish the defense abilities of the country significantly. It may be different in countries in which conscription is selective, that is, there must be a certain number of people in the military, during times of war (like the US during the Vietnam war), but no Western state, except Israel, has waged war with the use of conscripts in the last 40 years, the last such conflict being the American war in Vietnam.

Here is another possible argument for treating conscripts more harshly than physicians in the case of CO: one could argue that conscripts are far more likely to falsely invoke conscientious objection than physicians. At first glance, there seems to be little reason for a physician to seek to avoid participating in some medical procedure unless he has some moral objection to it, whereas any person has a strong self-interested reason to avoid being conscripted, namely that being a soldier is very dangerous. One could appeal to this difference as a basis for claiming that conscripts and soldiers must justify their refusal to participate while physicians do not. The evaluation of the weight of this allegation is problematic because—in its nature—it remains an empirical generalization unsub-stantiated by any specific research. But, as in the previous argument, with the exception of conscription during times of war, the situation seems to be exactly the opposite. There are no special prudential reasons for conscripts to avoid participating in the military during peacetime (usually, they just spend more time in an alternative service, which society usually treats as less prestigious). In contrast, there are very strong prudential reasons for physicians to refuse, since, for example, abortion in no way assists them in their professional or scientific careers—both because it is not cognitively interesting but may also be unwelcome, at least in some Catholic countries like Poland, by their superiors and many abortion clinics. Further, physicians who do not refuse are susceptible to antiabortion harassment.^16^

## 5 Conclusion

This paper argued that the legal regulations concerning the conscience clause for physicians should be at least similar to regulations applicable to conscripts or—in some countries—even to professional soldiers. It also argued that not only should the authenticity and relevance of a given judgment of conscience be evaluated but also whether this judgment is above some moral threshold of importance. Practically, it means that qualifications for conscientious objection in healthcare can be met only in some cases of destroying, killing, or helping in killing human organisms (abortion) but not in cases of “morning after” pills or other medical procedures.

## Footnotes

1 The judgment had been announced before the legal status of this institution got contested in December 2015, see: Sadurski 2019. The practical relevance of this judgment was undermined on October 22, 2020 when TK judged that “a high probability of a severe and irreversible fetal impairment or incurable illness endangering its [the fetus’s] life” as a legal condition for abortion is unconstitutional in Poland.

2 In this context, “lexical priority” means that, in a situation of conflict of rights, right to conscientious objection should take priority over any other rights, see Rawls 1999.

3 “Persons who are covered by social security insurance, and persons entitled by other regulations, are entitled right to free abortion in a public health care institution” (Sejm 1993).

4 For a history of discussions on moral conscience see Sorabji 2014.

5 “In fidelity to conscience, Christians are joined with the rest of men in the search for truth” (Paul VI 1965).

6 Compare with Harry G. Frankfurt’s 1987 discussion on wholeheartedness and integrity.

7 This lack of providers is the case of Podkarpackie voivodeship, located in the southeast of Poland with a population of 2.1 million and where there had been no official abortions in 2018 (see Rada Ministrów 2020).

8 For a review of Catholic views on this issue, see Coleman 2013. For other cases of disagreement among Catholic bioethicists, for example whether surgical separation of conjoined twins is permissible when one of them may die upon separation or whether it is permissible to withdraw medically provided nutrition and hydration from a patient in a persistent vegetative state, see Eberl 2017.

9 It is assumed that sexual orientation also belongs to the category “other,” as the patient ombudsperson has also acknowledged.

10 For a general discussion see Portmore 2021.

11 “[It] is necessary that each and every marriage act remain ordered *per se* to the procreation of human life” (Catechism 1993).

12 A similar argument may be applied to situations when a physician opposes offering some assisted reproductive technologies (ART) to LGBT couples, see Brummett 2018.

13 Cases of CO referring to the allocation of limited healthcare resources are not discussed here, see Wilkinson 2017.

14 A version of this view has been defended famously by Thomson 1971.

15 Eva LaFollette and Hugh LaFollette 2007 compare CO in healthcare with military refusals, and although they concentrate on pharmacists, the conclusion of their paper is similar to mine: “what objecting pharmacists expect—and not infrequently get—is far more than what conscientious objectors to war get or expect” (251).

16 Eighty-four percent of American clinics reported at least one form of antiabortion harassment in 2011 (Guttmacher Institute 2016). There are no easily available statistics for Poland on this matter, but there are regular press reports about such incidents.
